# The first complete mitochondrial genome of the genus *Echinolaelaps* reveals mitochondrial genome rearrangement type and evolution of Gamasida

**DOI:** 10.1017/S0031182023000367

**Published:** 2023-06

**Authors:** Bili Yuan, Gangxian He, Wenge Dong

**Affiliations:** Yunnan Provincial Key Laboratory for Zoonosis Control and Prevention, Institute of Pathogens and Vectors, Dali University, Dali, Yunnan 671000, China

**Keywords:** *Echinolaelaps echidninus*, Gamasida, mitochondrial genome, phylogeny, rearrangement

## Abstract

*Echinolaelaps echidninus* is a gamasid mite that is of medical and veterinary significance as parasites and vectors of disease agents, which can carry pathogens of zoonosis such as *Rickettsia tsutsugamushi*, *Rickettsia Q fever*, *Rickettsia mooseri*, *Rickettsia pox pathogens*, *Corynebacterium pseudotuberculosis* and *Leptospira*. At present, only single mitochondrial genes have been analysed for *E. echidninus* in the world, and no complete mitochondrial genome has been reported. However, information carried by a single gene is limited. Therefore, the complete mitochondrial genome of *E. echidninus* was determined for the first time by Illumina Hiseq X-Ten platform in this study. The mitochondrial genome is 15 736 bp in length and contains 13 protein-coding genes, 22 tRNA genes, 2 rRNA genes and a control region of 1561 bp in length. Codon analysis of 13 protein-coding genes revealed that UUU, UUA, AUU, AUA and AAU were the most frequently used, while *cox2* had the fastest evolutionary rate and *cob* the slowest. Comparative analysis of genome structure and breakpoint distances of the mitochondrial genomes of 23 species in 17 genera from 10 families of Gamasida deposited in GenBank revealed a novel gene arrangement type of the *E. echidninus* mitochondrial genome, and different degrees of rearrangement among different taxa of Gamasida. Phylogenetic analyses of Gamasida were performed using the maximum likelihood and Bayesian inference methods. *Echinolaelaps echidninus* was clustered with Dermanyssoidea and formed a more supportive sister group with *Varroa destructor.* This study provides novel insights into rearrangement patterns and evolution of mitochondrial genomes of Gamasida.

## Graphical abstract

**Figure:**
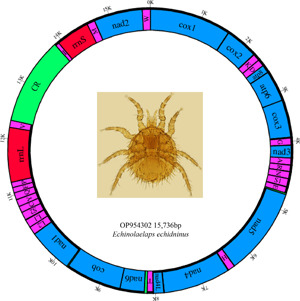

## Introduction

*Echinolaelaps echidninus* is a large worldwide mite belonging to Laelapidae, Gamasida, Parasitiformes, Acari (Deng, 1993). As early as 1929, H.E. Ewing separated the large mites from the genus *Laelaps* and defined 1 new genus *Echinolaelaps* (Wang, 1963) and then the Latin name for the mite was defined as *Echinolaelaps echidninus* Berlese, 1887. However, there are 2 Latin names of the mite due to differences in taxonomic opinion. Some researchers have been using *Laelaps echidninus* Berlese, 1887 (Mishra *et al*., 1970; Zhou *et al*., 2022), and others were using *E. echidninus* Berlese, 1887 (Pawar *et al*., 2016) or both (Ugbomoiko and Obiamiwe, 1991). Taxonomic relationships of Gamasida are also confusing, with different taxonomists holding different views. This paper follows the classification system of Krantz, 1978 (Deng,^1993^).

*Echinolaelaps echidninus* has a relatively wide range of hosts and can be parasitic on the body surface of most small mammals, among which *Rattus tanezemi*, *Rattus norvegicus* and *Mus musculus* are dominant hosts (Yang, 1992; Guo *et al*., 1996). *Echinolaelaps echidninus* is ovoviviparous and has a 4-stage life history: larva, first nymph, second nymph and adult. The larva does not feed, but the first nymph, second nymph and adult need to feed on blood, tissue fluids, wound exudate and other secretions of their hosts, making them an important medical mite that can directly damage host skin while feeding on animal and human blood, causing pruritus, maculopapular rash and even systemic reactions (Zhao, 2002; Chai *et al*., 2017). *Echinolaelaps echidninus* is also an important vector. Researchers have isolated zoonotic pathogens such as *Rickettsia tsutsugamushi*, *Rickettsia Q fever*, *Rickettsia mooseri*, *Rickettsia pox pathogens*, *Corynebacterium pseudotuberculosis* and *Leptospira* in *E. echidninus* (Deng, 1993; Krantz and Walter, 2010).

The typical mitochondrial genome of arthropods contains 37 genes, namely 22 tRNA genes, 13 protein-coding genes, 2 rRNA genes (*rrnL* and *rrnS*) and a variable length control region (CR) (Gissi *et al*., 2008). Because of its compact structure, smaller molecule (16–19 kb) than nuclear genome, 5–10 times faster evolution rate than nuclear genome and maternal inheritance, mitochondrial genome has become an important molecular marker for studying the origin of species, phylogenetic relationships between related species and within species in recent years (Yang *et al*., 2023). In the analysis of the mitochondrial genomes of Gamasida, which have been deposited in Genbank, the mitochondrial genomes of 5 species of mites in the families Diplogyniidae and Parasitidae (*Parasitus wangdunqingi*, *Parasitus fimetorum*, *Microdiplogynium* sp., *Quadristernoseta cf. longigynium* and *Quadristernoseta cf. intermedia*) retained the ancestral pattern of mitochondrial gene arrangement of arthropod, while other species show varying degrees of rearrangement (Thomas *et al*., 2011; Osuna-Mascaró *et al*., 2020; Zhang *et al*., 2021; Yang *et al*., 2022). However, there are multiple gene duplications in the mitochondrial genomes of *Euseius nicholsi* (2 of *trnM* and *trnF*) and *Metaseiulus occidentalis* (duplicated 18 genes). The genome composition of most arthropod mitochondrial genomes is very stable and the phenomenon of gene duplications or gene loss is rare (Jeyaprakash and Hoy, 2007; Li *et al*., 2019). In addition, researchers found tRNA truncation in Acariformes and the average tRNA length of 54.8 ± 1 bp in a previous study, and the average length of mitochondrial tRNAs in Parasitiformes is 62.0 ± 1.3 bp (Yuan *et al*., 2010). The length of Parasitiformes tRNA genes is longer than that of Acariformes, but shorter than the average length of arthropods tRNA genes (66 bp) (Jeyaprakash and Hoy, 2007), which prevents some tRNA genes from shaping the typical 4-armed cloverleaf secondary structure. This suggests that truncated tRNAs may occur in Parasitiformes.

Currently, the study of Gamasida is hampered by extreme body miniaturization, which has likely stifled the interest of taxonomists in this group and delayed the application of new sequencing technologies. This study filled a gap of the complete mitochondrial genome of the genus *Echinolaelaps*, and some novel insights into the rearrangement patterns and phylogeny of Gamasida are provided by the unique mitochondrial genome presented by *E. echidninus*.

## Materials and methods

### Specimen collection, morphological identification, DNA extraction and PCR amplification

**Figure 1.**
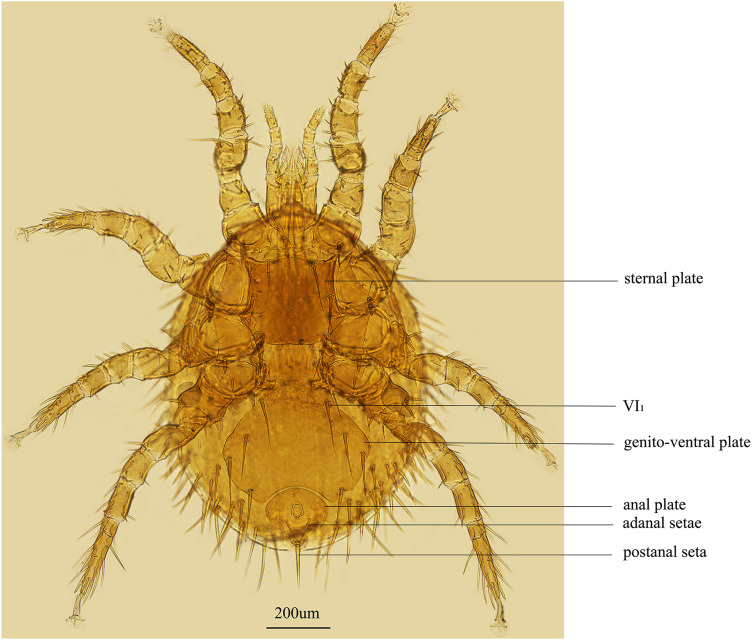
Morphological characteristics of *E. echidinus* from China.

*Echinolaelaps echidninus* were collected from the body surface of *Rattus tanezumi* (Muridae, Rodentia) in Yunnan Province, and all samples were stored at –80°C in a refrigerator for backup. The mite specimens and *R. tanezumi* were deposited in the Institute of Pathogens and Vectors, Dali University, China. The main reference for morphological identification of *E. echidninus* is the *volume 40 of the Economic insect fauna of China Fasc* (Deng, 1993). The essential distinguishing features of *E. echidninus* were that the 2 sides of the genito-ventral plate are extremely enlarged after VI_1_, the posterior margin was deeply concave and the distance between the genito-ventral plate and anal plate is small, showing a narrow groove. The anal plate is inverted pear-shaped, the front part is blunt and round and the rear end is sharp and narrow. The adanal setae lie behind the level of the back end of the anus and reach the base of the postanal seta. The postanal seta is thicker and longer than the adanal setae (Fig. 1).

Total DNA was extracted from individual mite with DNeasy Tissue Kit (QIAGEN, Germany). Ex Taq (Takara) and the conserved universal primers: bcdF01: 5′-CATTTTCHACTAAYCATAARGATATTGG-3′, bcdR04: 5′-TATAAACYTCDGGATGNCCAAAAAA-3′ (Dabert *et al*., 2010), 16Sbr: 5′-CCGGTCTGAACTCAGATCACGT-3′ and 16Sar: 5′-CGCCTGTTTAACAAAAACAT-3′ (Simon *et al*., 1994) were used to amplify 421 bp *cox1* gene and 300 bp *rrnL* gene of *E. echidninus*. The polymerase chain reaction (PCR) cycling conditions were 94°C for 3 min; 37 cycles of 94°C for 1 min, 46–54°C for 1 min, 72°C for 1 min; 72°C for 10 min. The long fragment-specific primers for *E. echidninus*, 43 *cox1*-*rrnL*-F2: 5′-CTGTTTATCCACCTTTGGCTGGAAG-3′ and 43 *cox1*-*rrnL*-R1: 5′-GCGACCTCGATGTTGAATTAATGAACC-3′, were designed with the sequences of *cox1* and *rrnL* short fragments to amplify the complete mitochondrial genome of *E. echidninus*. Amplification conditions were 98°C for 2 min; 34–40 cycles of 98°C for 10 s, 46–56°C for 30 s, 68°C for 5 min; 68°C for 10 min.

### Sequencing, assembly and annotation of mitochondrial DNA sequence

PCR products were purified with the Wizard SV Gel and PCR clean-up system (Promega) kit according to the manufacturer's instructions. Purified PCR products were sequenced directly with Illumina Hiseq X-Ten platform at Winner Biotech in Shanghai, China. The clean sequencing data of *E. echidninus* libraries were assembled with Geneious Prime 11.0 software (Kearse *et al*., 2012), tRNA genes were searched with tRNAscan SE (Chan *et al*., 2021) and ARWEN (Laslett and Canback, 2008), and protein-coding and rRNA genes were identified with Geneious Prime 11.0 software, BLAST and MITOS (Altschul *et al*., 1997; Bernt *et al*., 2013). The annotated mitochondrial genome sequence of *E. echidninus* was deposited in GenBank (accession number: OP954302).

### Rearrangement and phylogenetic analysis of the mitochondrial genome

The breakpoint distances between each of 2 taxa of Gamasida were compared using the CREx web server (Bernt *et al*., 2007) to analyse the extent of rearrangement of the mitochondrial genome (*M. occidentalis* and *E. nicholsi* were not calculated due to duplicated mitochondrial genes). Thirteen protein-coding genes of 24 complete mitochondrial genome sequences of 17 genera and 10 families of Gamasida, currently published in GenBank, were aligned using Mega 11 (Tamura *et al*., 2021). *Tachypleus tridentatus*, *Carcinoscorpius rotundicauda* and *Limulus polyphemus* were used as outgroups for phylogenetic analysis using the maximum likelihood (ML) method (Stamatakis, 2006) and Bayesian inference (BI) method (Ronquist and Huelsenbeck, 2003). In ML analysis, the GTR + I + G model constructs ML trees with 1000 bootstrap replicates. For BI analysis, 4 simultaneous Markov chains were run for 1 million generations, with tree sampling occurring every 1000 generations, and a burn-in of 25% of the trees in Mrbayes to phylogenetic analysis. Finally, the Figtree 1.4.4 program (http://tree.bio.ed.ac.uk/software/figtree/) was used to embellish the evolutionary tree.

## Result

### Base composition and genes distribution of the mitochondrial genome of *E. echidninus*

The complete mitochondrial genome of *E. echidninus* is 15 736 bp in length, was assembled by sequencing and contains 37 genes, namely 13 protein-coding genes, 22 tRNA genes, 2 rRNA genes (*rrnL* and *rrnS*) and a CR of 1561 bp in length. Base composition of the entire mitochondrial genome is A: 40.8%, C: 7.7%, G: 10.7%, T: 40.8%, with an AT content of 81.6%, AT-skew is 0 and GC-skew is 0.163. The lengths of *rrnL* and *rrnS* are 1079 and 714 bp, respectively, with *rrnL* on the N strand and *rrnS* on the J strand. Protein-coding and tRNA genes are on the J strand except for *nad5*, *nad4*, *nad4L*, *nad1*, *trnL_1_*, *trnL_2_*, *trnS_2_*, *trnH*, *trnC*, *trnQ*, *trnY*, *trnF*, *trnP* and *trnV*, which were located in the N strand (Table 1).

**Table 1.** Distribution of the *E. echidninus* complete mitochondrial genome

Name	Position	Length (bp)	Strand	Start codons	Stop codons
*cox1*	1–1575	1575	J	ATA	TAA
*cox2*	1581–2276	696	J	ATG	TAA
*trnK*	2283–2346	64	J		
*trnD*	2348–2409	62	J		
*atp8*	2410–2523	114	J	ATT	TAG
*atp6*	2520–3185	666	J	ATA	TAA
*cox3*	3185–3982	798	J	ATG	TAA
*trnG*	3966–4027	62	J		
*nad3*	4025–4360	336	J	ATA	TAA
*trnA*	4361–4421	61	J		
*trnR*	4424–4486	63	J		
*trnN*	4484–4547	64	J		
*trnS_1_*	4546–4608	63	J		
*trnE*	4615–4677	63	J		
*nad5*	4704–6377	1674	N	ATT	TAA
*trnH*	6381–6442	62	N		
*nad4*	6452–7756	1305	N	ATG	TAA
*nad4L*	7764–8060	297	N	ATT	TAA
*trnT*	8053–8112	60	J		
*nad6*	8162–8599	438	J	ATA	TAA
*cob*	8602–9708	1107	J	ATG	TAA
*nad1*	9730–10 641	912	N	ATT	TAA
*trnL_2_*	10 639–10 699	61	N		
*trnL_1_*	10 701–10 765	65	N		
*trnS_2_*	10 773–10 839	67	N		
*trnC*	10 840–10 889	50	N		
*trnY*	10 890–10 954	65	N		
*trnQ*	10 953–11 020	68	N		
*trnF*	11 021–11 084	64	N		
*trnP*	11 085–11 147	63	N		
*rrnL*	11 148–12 226	1079	N		
*trnV*	12 227–12 288	62	N		
CR	12 289–13 849	1561	J		
*trnI*	13 850–13 917	68	J		
*rrnS*	13 918–14 631	714	J		
*trnM*	14 632–14 693	62	J		
*nad2*	14 696–15 673	978	J	ATA	TAA
*trnW*	15 674–15 736	63	J		

### Analysis of tRNA and protein-coding genes in the mitochondrial genome of *E. echidninus*

**Figure 2.**
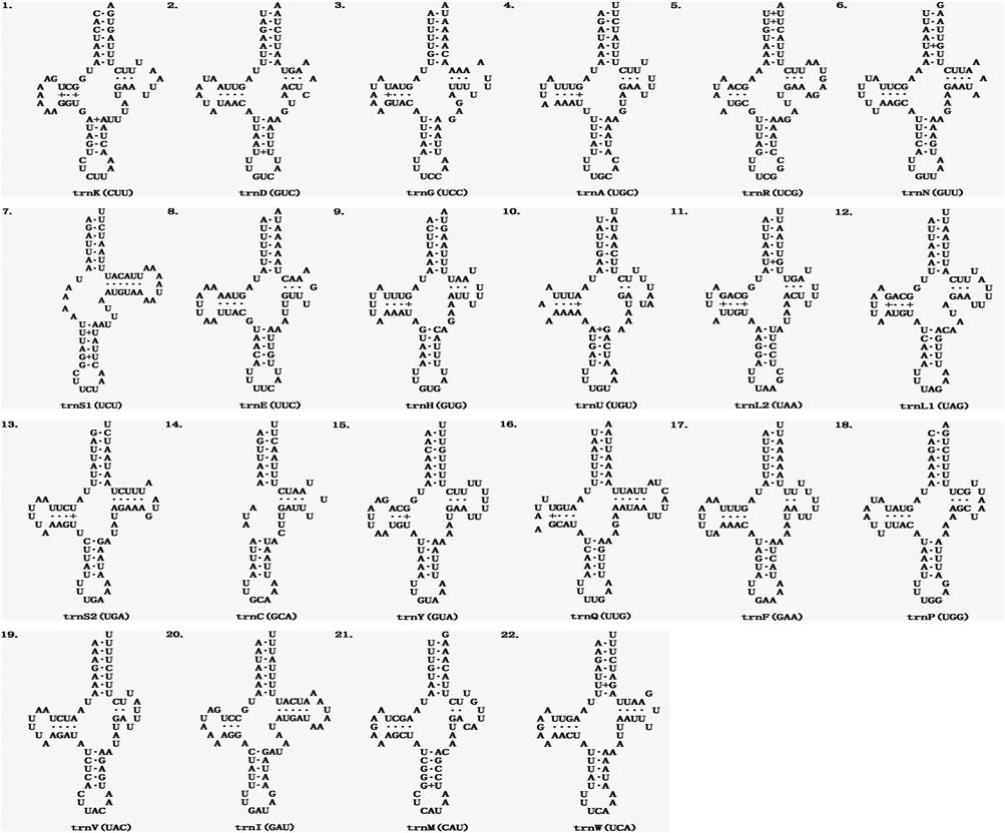
Secondary structure of the 22 mitochondrial tRNAs of *E. echidninus.*

The average length of 22 mitochondrial tRNA genes of *E. echidninus* was 62 ± 0.8 bp, of which *trnC* gene (50 bp in length) was the shortest, *trnQ* and *trnI* were the longest (68 bp in length). Except for *trnC* and *trnS_1_*, which are missing the D-arm, all the tRNAs have the typical 4-armed cloverleaf secondary structure (Fig. 2). The anticodon of *trnK* is CUU, which does not preserve the universal anticodon of the arachnid mitochondrial tRNA (*trnK*: UUU), while the rest of tRNAs preserve the universal anticodon of the arachnid mitochondrial tRNA. The length of 13 protein-coding genes in order is *nad5>cox1>nad4>cob>nad2>nad1>cox3>cox2>atp6>nad6>nad3>nad4L>atp8*. There were 3632 amino acids encoded in the complete mitochondrial genome of *E. echidninus*. *Cox1*, *atp6*, *nad3*, *nad6*, *nad2* have ATA as the start codon, c*ox2*, *cox3*, *nad4*, *cob* have ATG as the start codon and *atp8*, *nad5*, *nad4*, *nad1* have ATT as the start codon. All protein-coding genes have complete stop codons, except *atp8* which uses TAG as a stop codon, and the remaining 12 protein-coding genes use TAA as a stop codon (Table 1).

**Figure 3.**
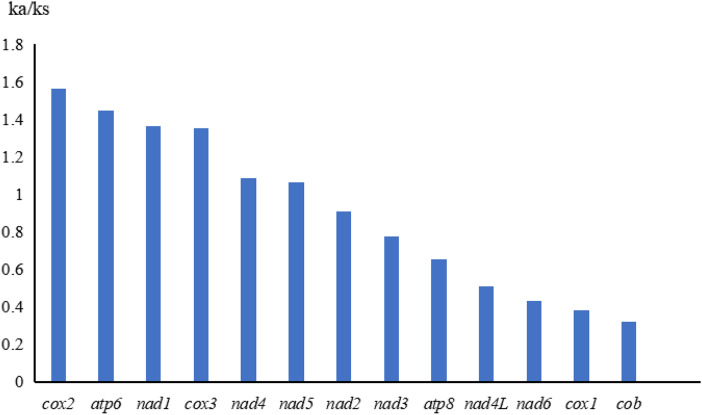
Comparison of non-synonymous and synonymous evolutionary rates (Ka/Ks) of 13 protein-coding genes in *E. echidninus* using the *Tachypleus tridentatus* as outgroup.

The relative synonymous codon usage (RSCU) was analysed for the mitochondrial genome of *E. echidninus* (Table 2). UUU, UUA, AUU, AUA and AAU were the most frequently used (>150 times). Twenty-eight codons of UUA, AGA, UCU, CCU, GCU, etc., are preference codons, and the RSCU was >1. The codons of Ile, Leu, Phe and Ser were the most frequently used (1780 times in total), accounting for 49.0% of the total number of codons for uses. Using *T. tridentatus* as an outgroup, the evolutionary rate of 13 protein-coding genes was calculated and analysed with DnaSP6 (Rozas *et al*., 2017). Among the 13 protein-coding genes, the ratio of non-synonymous evolutionary rate to synonymous evolutionary rate (Ka/Ks) for *cox2, atp6, nad1, cox3, nad4 and nad5* was >1, while the ratio of non-synonymous evolutionary rate to synonymous evolutionary rate (Ka/Ks) for the rest of the protein-coding genes was <1. The evolutionary rates of 13 protein-coding genes were *cox2>atp6>nad1>cox3>nad4>nad5>nad2>nad3>atp8>mad4L>nad6>cox1>cob* (Fig. 3).

**Table 2.** Codon usage in protein-coding genes in the *E. echidninus* complete mitochondrial genome

Amino acid	Codon	Number of uses	RSCU	Amino acid	Codon	Number of uses	RSCU
Phe	**UUU**	**435**	**1**.**89**	Ser	UCU	137	**2**.**92**
	UUC	25	0.11		UCC	7	0.15
Leu	**UUA**	**408**	**4**.**65**		UCA	78	**1**.**66**
	UUG	32	0.37		UCG	4	0.09
	CUU	54	0.62	Pro	CCU	82	**3**.**07**
	CUC	4	0.05		CCC	4	0.15
	CUA	26	0.30		CCA	19	0.71
	CUG	2	0.02		CCG	2	0.07
Ile	**AUU**	**404**	**1**.**93**	Thr	ACU	72	**2**.**46**
	AUC	15	0.07		ACC	4	0.14
Met	**AUA**	**234**	**1**.**82**		ACA	41	**1**.**40**
	AUG	23	0.18		ACG	0	0.00
Val	GUU	96	**2**.**31**	Ala	GCU	69	**2**.**71**
	GUC	5	0.12		GCC	4	0.16
	GUA	58	**1**.**40**		GCA	29	**1**.**14**
	GUG	7	0.17		GCG	0	0.00
Tyr	UAU	147	**1**.**86**	Cys	UGU	30	**1**.**88**
	UAC	11	0.14		UGC	2	0.13
TER	UAA	12	**1**.**85**	Trp	UGA	70	**1**.**75**
	UAG	1	0.15		UGG	10	0.25
His	CAU	68	**1**.**86**	Arg	CGU	29	**2**.**47**
	CAC	5	0.14		CGC	0	0.00
Gln	CAA	39	**1**.**73**		CGA	17	**1**.**45**
	CAG	6	0.27		CGG	1	0.09
Asn	**AAU**	**182**	**1**.**88**	Ser	AGU	26	0.55
	AAC	12	0.12		AGC	1	0.02
Lys	AAA	99	**1**.**69**		AGA	120	**2**.**56**
	AAG	18	0.31		AGG	2	0.04
Asp	GAU	73	**1**.**78**	Gly	GGU	70	**1**.**72**
	GAC	9	0.22		GGC	2	0.05
Glu	GAA	85	**1**.**72**		GGA	71	**1**.**74**
	GAG	14	0.28		GGG	20	0.49

Highlight codons with RSCU > 1 in bold.

### Arrangement pattern and rearrangement degree of the mitochondrial genome of *E. echidninus*

Among the complete mitochondrial genomes of Gamasida that have been studied so far, the complete mitochondrial genome of *E. echidninus* shows a novel arrangement pattern. Except that *trnC*, *trnY*, *trnQ*, *trnF*, *trnP* and *trnI* were translocated, and *trnS_2_* and *rrnS* were translocated and inverted, the remaining 13 protein-coding genes and tRNA genes still retained the ancestral pattern of mitochondrial gene arrangement of arthropods. Because of the rearrangement of *rrnS* gene, the ancestral arthropod ‘*rrnL*-*trnV*-*rrnS*’ gene cluster is no longer retained by *E. echidninus*. By breakpoint distance analysis (Table 3), comparing with that of the ancestral arthropod (as the presentative of *T. tridentatus*), the breakpoint distance of the mitochondrial genome of *E. echidninus* is 13; the breakpoint distances of *Amblyseius tsugawai* and *Amblyseius swirskii* are the highest (33); the breakpoint distances of *Macrocheles muscaedomesticae* and *Stylochyrus rarior* are the lowest (7). The breakpoints of species in the same family are similar, with certain differences between different families.

**Table 3.** Breakpoint distance analysis between species of Gamasida

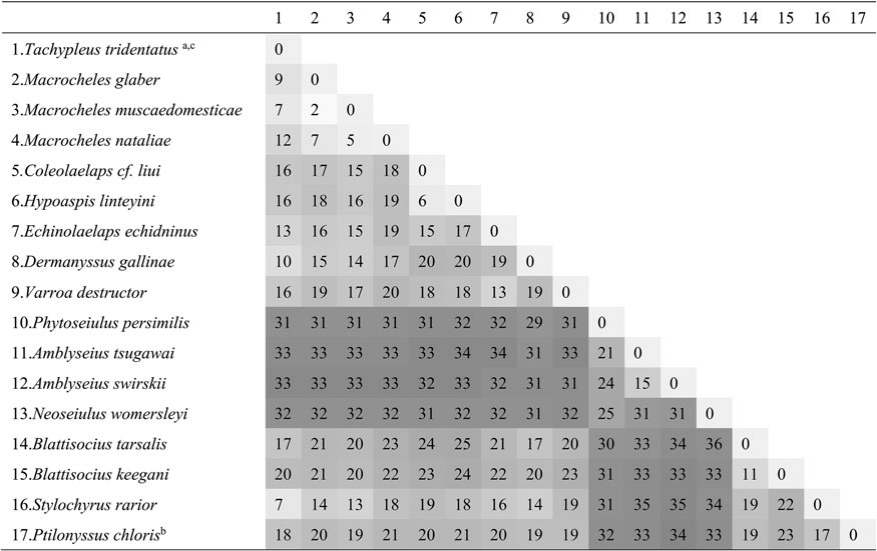

^a^“1.*Tachypleus tridentatus*” (as the presentative of ancestral arthropod) retained the ancestral pattern of mitochondrial gene arrangement of arthropod.

^b^The mitochondrial gene arrangement patterns of *Ptilonyssus chloris* and *Tinaminyssus melloi* are consistent. Two species are represented by “17.*Ptilonyssus chloris*” in the table.

^c^The mitochondrial gene of 5 species of mites in Diplogyniidae and Parasitidae (*Parasitus wangdunqingi*, *Parasitus fimetorum*, *Microdiplogynium* sp., *Quadristernoseta cf. longigynium* and *Quadristernoseta cf. intermedia*) retained the ancestral pattern of mitochondrial gene arrangement of arthropod. Five species are represented by “1.*Tachypleus tridentatus*” in the table.

### Phylogenetic relationships of *E. echidninus*

**Figure 4.**
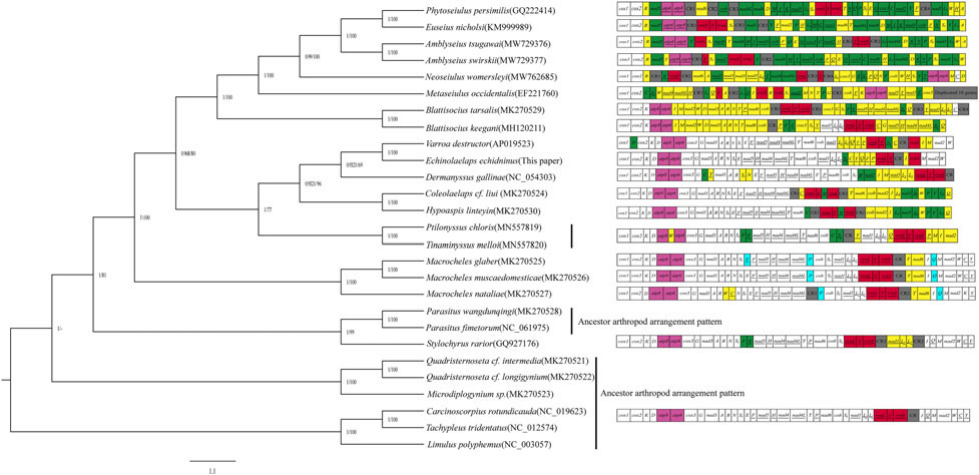
Phylogenetic relationships of Gamasida were inferred from the nucleotide sequences of 13 protein-coding genes using the Bayesian inference (BI) and maximum likelihood (ML) methods. The posterior probabilities for BI (left) and bootstrap support values for ML (right) are shown on the corresponding nodes in the identical topology of BI and ML tree. On the right side of the picture are the mitochondrial genomic arrangement patterns of the species, the different colours have different meanings (yellow: translocations; blue: inversions; green: translocations and inversions; red marks the ‘*rrnL-V-rrnS*’ gene cluster; pink marks the atp8 and atp6 genes; grey marks the control region and the 18 duplicated genes in the *Metaseiulus occidentalis*).

For the phylogenetic analysis of Gamasida using the ML (Stamatakis, 2006) and BI methods (Ronquist and Huelsenbeck, 2003), *T. tridentatus*, *C. rotundicauda* and *L. polyphemus* were used as the outgroup and the 13 protein-coding genes of 24 species in 17 genera and 10 families of the Gamasida as the ingroup. The phylogenetic tree of Gamasida is divided into 2 main branches (Fig. 4). The first branch consists of 3 species of 2 genera in the family Diplogyniidae located at the base of the phylogenetic tree, and the second branch contains the largest number of species, which consist of 21 species of 15 genera and 9 families. *Echinolaelaps echidninus* is located in the second branch, clustered with the species of Dermanyssoidea and formed a more supportive sister group with *Varroa destructor* in this study.

## Discussion

The mitochondrial genome of *E. echidninus* was reported for the first time in this study. It is 15 736 bp in length and contains 37 genes and 1 CR typical of mitochondrial genomes. AT average content of the *E. echidninus* mitochondrial genome was 81.6%, with AT-skew of 0 and GC-skew of 0.163. The AT-skew of the mite is 0, which is more special in the mitochondrial genome of Acari. In general, due to the directional mutation pressure and asymmetric replication process, the AT skew of the J strand of the mitochondrial genome of most mites is positive, while the GC skew is negative (Hassanin *et al*., 2005). Does a zero value for the J strand AT skew of *E. echidninus* predict low directional mutational pressure on its mitochondrial genome and/or a relatively short period of time for the mitochondrial double strand to remain in a single strand? Among the other species of Gamasida, we found that *V. destructor*, *E. nicholsi*, *Phytoseiulus persimilis*, *A. tsugawai* and *A. swirskii* showed negative AT-skew and positive GC-skew, while *Coleolaelaps cf. liui*, *Blattisocius keegani* and *Hypoaspis linteyini* showed negative AT-skew and GC-skew. The remaining mites maintained base skew characteristic of the mitochondrial genome of Acari. Analysing from parasitic host, living environment, life history and other factors, AT skew value does not seem to show significant species specificity, so the mechanisms responsible for differences in AT skew between the same genera need to be further explored in the next studies.

The 2 rRNAs of the ancestral arthropods are both located in the N strand and contain an *rrnL*-*trnV*-*rrnS* gene cluster, but *rrnS* is located in the J strand while *rrnL* is located in the N strand in the *E. echidninus* mitochondrial genome, due to a translocation and inversion of *rrnS*. This phenomenon is relatively rare in the mitochondrial genomes of Gamasida, and is currently only found in *V. destructor* from Japan (Harada *et al*., 2020) and *E. echidninus* studied here. The average length of mitochondrial tRNAs was 54.8 ± 1 bp in the Acariformes and 62.0 ± 1.3 bp in the Parasitiformes (Yuan *et al*., 2010). The average length of the 22 tRNA genes of *E. echidninus* is 62 ± 0.8 bp, with *trnC* (50 bp) being the shortest and *trnQ* (68 bp) and *trnI* (68 bp) being the longest. All tRNAs have typical cloverleaf secondary structure, with the exception of *trnC* and *trnS_1_*, which lack the D-arm (Fig. 2). The absence of the D-arm of *trnS_1_* is an ancestral feature of the Acari mitochondrial genome (Wolstenholme, 1992). The missing D-arm of *trnC* is reported for the first time in *Echinolaelaps* and may be of some reference value for succeeding studies. Typically, mitochondrial tRNAs in mites retain the universal anticodons of Arachnida mitochondrial tRNAs, but *E. echidninus* studied here show *trnK* (UUU to CUU) and most of the species of Gamasida show *trnK* (UUU to CUU) and/or *trnS_1_* (UCU to GCU), which may be a synapomorphy of Gamasida.

The start codons of 13 protein-coding genes are ATN and the stop codons are TAA or TAG, encode a total of 3632 amino acids. The frequency of UUU, UUA, AUU, AUA and AAU was considered to be high (>150 times) according to the analysis of synonymous codon usage (Table 2). Twenty-eight codons, including UUA, AGA, UCU, CCU and GCU, were preferred codons, with RSCU >1. The codons for Ile, Leu, Phe and Ser were the most frequently used (1780 times in total), accounting for 49.0% of the total codon usage. Codon preference can reveal the proximity of species relatedness and is used in the taxonomic study of species (Sharp and Li, 1986; Kokate *et al*., 2021), so to a certain extent codon preference can reveal the taxonomic relationships of species. Based on the analysis of evolutionary rates of 13 protein-coding genes in *T. tridentatus* (Fig. 3), *cox2*, *atp6*, *nad1*, *cox3*, *nad4* and *nad5*, the ratio of non-synonymous evolutionary rate to synonymous evolutionary rate (Ka/Ks) was >1, demonstrating that these 6 protein-coding genes are subject to positive selection, and because of this positive selection, the species are able to continuously improve their adaptability to the environment. The ratio of non-synonymous to synonymous evolutionary rates (Ka/Ks) for the remaining protein-coding genes is <1, suggesting that these protein-coding genes are subject to negative selection, and the effect of negative selection is to restrict mutations in mitochondrial genes so that oxidative phosphorylation-related protein functions in mitochondria are performing their functions in a consistent manner (Hurst, 2002; Wang *et al*., 2014).

In Gamasida, with the exception of Parasitidae and Diplogyniidae, which retain the ancestral arthropod mitochondrial genome arrangement pattern, other species show rearrangements with varying degrees. The breakpoint distance analysis (Table 3) (*M. occidentalis* and *E. nicholsi* were not calculated due to duplicated mitochondrial genes) revealed that the breakpoint distances of *A. tsugawai* and *A. swirskii* are the highest (33) compared to the ancestral arthropods, while the breakpoint distances of *M. muscaedomesticae* and *S. rarior* are the lowest (7). The phylogenetic analysis of Gamasida (Fig. 4) revealed that *E. echidninus* and *V. destructor*, *A. tsugawai* and *A. swirskii*, *Blattisocius tarsalis* and *B. keegani*, *C. cf. liui* and *H. linteyini*, *Ptilonyssus chloris* and *Tinaminyssus mello*, *Macrocheles glaber* and *M. muscaedomesticae*, *P. wangdunqingi* and *P. fimetorum*, *Quadristernoseta cf. longigynium* and *Quadristernoseta cf. intermedia* formed sister branches and the breakpoint distances between each sister branch were 13, 15, 11, 6, 0, 2, 0, 0, respectively. This suggests a different degree of mitochondrial genome rearrangement within species that form sister branches, with more closely related species maintaining a relatively constant pattern of gene arrangement (e.g. *P. chloris and T. mello*, *M. glaber* and *M. muscaedomesticae*, *P. wangdunqingi* and *P. fimetorum*, *Q. cf. longigynium* and *Q. cf. intermedia*), but also rapid gene rearrangements in a relatively short evolutionary time (e.g. *E. echidninus* and *V. destructor*, *A. tsugawai* and *A. swirskii*, *B. tarsalis* and *B. keegani*, *C. cf. liui* and *H. linteyini*). However, the breakpoint distances of mitochondrial genome between species that form sister branches are smaller (<15) compared to other species of Gamasida. The similarity of breakpoint distances between species of the same family or genus may be a typical feature of specific taxa (e.g. Phytoseiidae, Macrochelidae). Phylogenetic analysis supported gene rearrangement breakpoint analyses within Gamasida: *E. echidninus* was sister of *V. destructor* and showed a moderate number of breakpoints (13). Therefore, mitochondrial genome rearrangements may occur regularly with evolutionary divergence in Gamasida. There are certain similarities in rearrangement patterns and breakpoints distance in closely related taxonomic level.

*Echinolaelaps echidninus* belongs to Dermanyssoidea, so their breakpoint distances and mitochondrial genome arrangement patterns are similar to those of other species in Dermanyssoidea. Whilst 13 protein-coding genes retained the ancestral arthropod arrangement order, tRNA and rRNA show variant degrees of rearrangement. The analysis of the phylogenetic tree (Fig. 4) revealed that *E. echidninus* clustered into a taxon with other species of Dermanyssoidea (*H. linteyini*, *C. cf. liui*, *V. destructor*, *Dermanyssus gallinae*, *P. chloris*, *T. mello*), but did not form a sister branch with other species of Laelapidae (*H. linteyini*, *C. cf. liui*) that were more closely related morphologically, instead of forming a sister group with *V. destructor*, indicating a close kinship between *V. destructor* and *E. echidninus* at the molecular level. This is not only the concentration of the complete mitochondrial genome arrangement pattern, but also is a further confirmation at the phylogenetic level. In terms of morphological classification, both *E. echidninus* and *V. destructor* belong to the large gamasid mite, but they belong to different families, with morphological characteristics of *E. echidninus* being more similar to those of species in Laelapidae. However, phylogenetic analyses at the molecular level have shown that *E. echidninus* is more closely related to *V. destructor*. Casanueva suggested that Varroidae was synonymized into Laelapidae (Casanueva, 1993), which is consistent with the results of the phylogenetic tree in this paper. It seems to suggest that a single morphological classification is not sufficient in the taxonomic study of Acari and that strong molecular evidence is needed to provide a more complete explanation of their taxonomic and phylogenetic relationships.

## Conclusion

Structure and evolution of the *E. echidninus* complete mitochondrial genome were reported for the first time in this study. It provides novel insights into rearrangement patterns and evolution of the complete mitochondrial genomes of Gamasida. The complete mitochondrial genome of the mite has 37 genes typical of metazoans, including 13 protein-coding genes, 22 tRNA genes and 2 rRNA genes. It is unusual for the *E. echidninus* complete mitochondrial genome structure with zero AT-skew and positive GC-skew, unlike the complete mitochondrial genome structure of Gamasida. The mitochondrial gene arrangement patterns and breakpoint distances indicated that the complete mitochondrial genomes of *E. echidninus* show a novel arrangement pattern. Phylogenetic relationships analysis of Gamasida showed that *E. echidninus* is located in the second branch and clustered into a taxon with other species of Dermanyssoidea (*H. linteyini*, *C. cf. liui*, *V. destructor*, *D. gallinae*, *P. chloris*, *T. mello*), the close kinship between *V. destructor* and *E. echidninus*. To obtain a more reliable phylogenetic tree, we still need to collect more representative species of Gamasida, to sequence the complete mitochondrial genomes of more species, and to study evolutionary mechanisms and rearrangement patterns of Gamasida in depth.

## Data Availability

All data generated or used during the study appear in the submitted article.
